# The Existing Drug Nifuroxazide as an Antischistosomal Agent: *In Vitro*, *In Vivo*, and *In Silico* Studies of Macromolecular Targets

**DOI:** 10.1128/spectrum.01393-23

**Published:** 2023-07-06

**Authors:** Vinícius Roquini, Ana C. Mengarda, Rayssa A. Cajas, Milene F. Martins-da-Silva, Julia Godoy-Silva, Gustavo A. Santos, Maria Cristina C. Espírito-Santo, Thais F. A. Pavani, Vanusa A. Melo, Maria C. Salvadori, Fernanda S. Teixeira, Daniela G. G. Rando, Josué de Moraes

**Affiliations:** a Research Center on Neglected Diseases, Guarulhos University, Guarulhos, São Paulo, Brazil; b Laboratory of Immunopathology of Schistosomiasis (LIM-06), Department of Infectious and Parasitic Diseases, Faculty of Medicine, University of São Paulo, São Paulo, São Paulo, Brazil; c Laboratory of Helminthology, Institute of Tropical Medicine, University of São Paulo, São Paulo, São Paulo, Brazil; d Biological Chemistry Post-Graduate Course, Institute of Environmental, Chemical and Pharmaceutical Sciences, Federal University of São Paulo, Diadema, São Paulo, Brazil; e Institute of Physics, University of São Paulo, São Paulo, São Paulo, Brazil; f Chemico-Pharmaceutical Research Group, Institute of Environmental, Chemical and Pharmaceutical Sciences, Federal University of São Paulo, Diadema, São Paulo, Brazil; National University of Singapore

**Keywords:** antibiotic, anthelmintic agent, drug discovery, drug repurposing, medicinal chemistry, parasitic diseases, schistosomiasis

## Abstract

Schistosomiasis is a parasitic disease that afflicts approximately 250 million people worldwide. There is an urgent demand for new antiparasitic agents because praziquantel, the only drug available for the treatment of schistosomiasis, is not universally effective and may derail current progress toward the WHO goal of eliminating this disease as a public health problem by 2030. Nifuroxazide (NFZ), an oral nitrofuran antibiotic, has recently been explored to be repurposed for parasitic diseases. Here, *in vitro*, *in vivo*, and *in silico* studies were conducted to evaluate the activity of NFZ on Schistosoma mansoni. The *in vitro* study showed significant antiparasitic activity, with 50% effective concentration (EC_50_) and 90% effective concentration (EC_90_) values of 8.2 to 10.8 and 13.7 to 19.3 μM, respectively. NFZ also affected worm pairing and egg production and induced severe damage to the tegument of schistosomes. *In vivo*, a single oral dose of NFZ (400 mg/kg of body weight) to mice harboring either prepatent or patent S. mansoni infection significantly reduced the total worm burden (~40%). In patent infection, NFZ achieved a high reduction in the number of eggs (~80%), but the drug caused a low reduction in the egg burden of animals with prepatent infection. Finally, results from *in silico* target fishing methods predicted that serine/threonine kinases could be one of the potential targets for NFZ in S. mansoni. Overall, the present study revealed that NFZ possesses antischistosomal properties, mainly in terms of egg burden reduction in animals with patent S. mansoni infection.

**IMPORTANCE** The increasing recognition of the burden imposed by helminthiasis, associated with the limited therapeutic arsenal, has led to initiatives and strategies to research and develop new drugs for the treatment of schistosomiasis. One of these strategies is drug repurposing, which considers low-risk compounds with potentially reduced costs and shorter time for development. In this study, nifuroxazide (NFZ) was evaluated for its anti-Schistosoma mansoni potential through *in vitro*, *in vivo*, and *in silico* studies. *In vitro*, NFZ affected worm pairing and egg production and induced severe damage to the tegument of schistosomes. *In vivo*, a single oral dose of NFZ (400 mg/kg) to mice harboring either prepatent or patent S. mansoni infection significantly reduced the total worm burden and egg production. *In silico* investigations have identified serine/threonine kinases as a molecular target for NFZ. Collectively, these results implied that NFZ might be a potential therapeutic candidate for the treatment of schistosomiasis.

## INTRODUCTION

Schistosomiasis, a parasitic disease caused by a blood fluke of the genus *Schistosoma*, is a debilitating disease with a serious global burden (1). It is a chronic disease of poverty characterized by pain and disability that, collectively, exacerbates the already compromised situation of health care in tropical and subtropical areas. Estimates show that at least 230 million people are affected with schistosomiasis and approximately 10% of the world population is at risk of infection (2). Schistosoma mansoni, one of the three major human species, is responsible for public health problems in Africa, the Middle East, the Caribbean, and South America. The morbidity associated with schistosome infection is driven almost entirely by the parasite’s massive egg output, causing hepatosplenomegaly, liver fibrosis, and ascites; in severe cases, S. mansoni infection can be fatal (3).

Since no effective vaccine against schistosomiasis is available, the Word Health Organization (WHO) strategy for schistosomiasis morbidity and transmission focuses on large-scale treatment (preventive chemotherapy) with praziquantel (PZQ). For example, in 2019, an estimate of at least 236.6 million people required preventive treatment for schistosomiasis (2). Although schistosomiasis control has been generally successful across many countries, numerous persistent hot spots remain, and several studies reported the reduced efficacy of praziquantel (4–7). The WHO revised neglected tropical disease (NTD) roadmap targets the elimination of schistosomiasis as a public health problem in all areas of endemicity by 2030 (8). However, any possibility of selection of the parasites for praziquantel resistance or low sensitivity may hamper the 2030 target of global disease elimination. As a result, the WHO recognizes the importance of initiatives for the development of safe, affordable, and effective drugs for schistosomiasis (9–11).

Strategies that have been applied successfully to expedite the discovery process for drugs against parasitic diseases include drug repurposing (12, 13). Known as “new uses for old drugs,” drug repurposing is an approach for identifying new uses for approved or investigational drugs that are outside the scope of the original medical indication. Increasingly, several research groups around the world, including ours, are considering this strategy to alleviate the dilemma of drug shortages, including in the search for new antischistosomal agents (14–18).

Nitrofuran derivatives have been used for decades to treat infectious diseases. In the early 1960s, nitrofurans were used to treat patients with schistosomiasis in China, but toxicity and suboptimal activity led to the abandonment of these compounds (19). Although some nitrofuran drugs have been withdrawn from the market, most of them remain used today in human or veterinary medicine. Nifuroxazide (NFZ) is an oral nitrofuran antibiotic that has been used successfully for many decades for the treatment of infectious colitis and diarrhea. The drug has proved to be well tolerated and safe, and it is available in several countries worldwide. NFZ was also explored as a potential antiparasitic agent, particularly to be repurposed for diseases caused by protozoa (for review see reference 20 and 21). However, studies using NFZ against parasitic worms are scarce. As part of our continuous effort to identify drugs with anthelmintic properties, in this study, we evaluated the antiparasitic activity of NFZ against S. mansoni. Based on the phenotypic assay, NFZ was first tested against adult parasites *ex vivo*, and subsequently, the 50% effective concentration (EC_50_) and 90% effective concentration (EC_90_) values were determined. The motility and morphology of the schistosomes were also monitored using light and scanning electron microscopy. Furthermore, NFZ was administered orally in a murine model infected with either immature (prepatent infection) or adult (patent infection) S. mansoni to characterize the full spectrum of activity of this drug. Finally, target fishing simulations were applied to predict the potential targets and pathways for NFZ in schistosomes.

## RESULTS

### NFZ alters the viability of adult schistosomes *in vitro*.

The *in vitro* antiparasitic potential of NFZ was assessed initially at different concentrations on S. mansoni worm pairs (male and female). For comparison, the known antischistosomal drug PZQ was used as a positive control, whereas vehicle-treated parasites (0.5% DMSO, representing the highest concentration of solvent) were used as a negative control. *In vitro* EC_50_ and EC_90_ values (72 h of incubation) of NFZ and PZQ obtained against adult worms of S. mansoni are summarized in Table 1. EC_50_ values of NFZ were 8.28 and 13.79 μM at 72 h against male and female worms, respectively. The EC_90_ values of NFZ were above 10 and 15 μM for male and female schistosomes, respectively. In comparison, PZQ has an EC_50_ value below 1 μM against adult worm pairs. As expected, 0.5% DMSO had no toxic effect on adult S. mansoni.

**TABLE 1:** *In vitro* activities of nifuroxazide and praziquantel against adult S. mansoni and cytotoxicity^*a*^

Drug	Schistosome results				Vero cell CC_50_	SI
	EC_50_		EC_90_			
	Male	Female	Male	Female		
NFZ	8.28 (6.4–11.5)	13.79 (11.4–16.7)	10.84 (9.18–12.17)	19.32 (17.43–23.57)	>200	>14
PZQ	0.63 (0.56–0.72)	0.81 (0.68–0.96)	0.97 (0.89–1.19)	1.21 (1.02–1.38)	>200	>246

a S. mansoni adult worms were exposed for 72 h to the tested compounds to calculate the EC_50_ and EC_90_ values. Data were calculated from three experiments, and each experiment was performed with three replicates. EC_50_, 50% effective concentration against adult worm pairs (male and female); CC_50_, 50% cytotoxic concentration against Vero cells; SI, selectivity index. Values are shown with 95% confidence intervals in parentheses.

**FIG 1:**
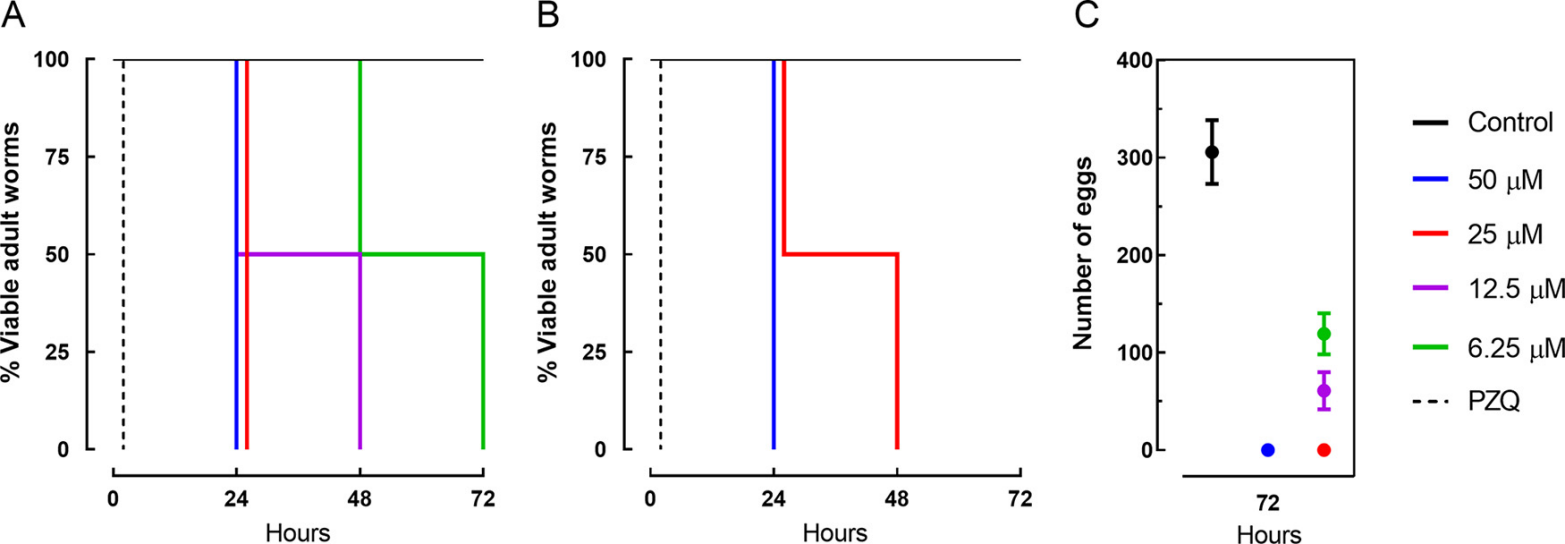
Viability and egg output of adult S. mansoni worms during exposure to nifuroxazide (NFZ) and praziquantel (PZQ). Adult worm pairs were obtained from animals by perfusion at 42 days after infection. Each concentration was tested at least in triplicate, with the highest concentration of DMSO serving as the control. Male (A) and female (B) schistosomes were monitored for up to 72 h, and results are expressed as the percent mortality recorded by Kaplan-Meier survival curves. Eggs released by paired adult worms exposed to NFZ (C). Data are presented as the mean ± SD from three independent experiments (*n *= 3). Control, drug-free medium; PZQ, praziquantel at 2 μM.

Assays regarding the survival times of adult worm pairs were also performed with NFZ to understand the kinetics and mode of action. As shown in Fig. 1, NFZ induced mortality in a time- and concentration-dependent manner. Similar to the results shown in Table 1, the antiparasitic assay revealed that male schistosomes are more susceptible to NFZ (Fig. 1A) than female worms (Fig. 1B). For example, when adult worm pairs were exposed to NFZ at a concentration of 25 μM for 48 or 72 h, 100% mortality was observed only for male parasites. Throughout the incubation period, parasites in the negative-control group (0.5% DMSO) remained viable. In contrast, PZQ caused the mortality of all worms immediately.

### NFZ affects S. mansoni egg output *in vitro*.

The capacity of NFZ to affect worm pairing and egg production was evaluated, and the number of eggs within 72 h is shown in Fig. 1C. During the assay, worm couples in the negative-control group moved actively and remained paired throughout the treatment. All adult worm pairs separated after incubation with NFZ at 25 and 50 μM, and they remained separated throughout the incubation period. Thus, a complete lack of oviposition was observed when adult schistosomes were exposed to NFZ at a concentration of ≥25 μM. At a concentration of 12.5 μM, the helminths remained coupled, but the number of eggs was significantly reduced compared with that of control worms (*P < *0.001).

### NFZ induces severe damage to the tegument of schistosomes.

**FIG 2:**
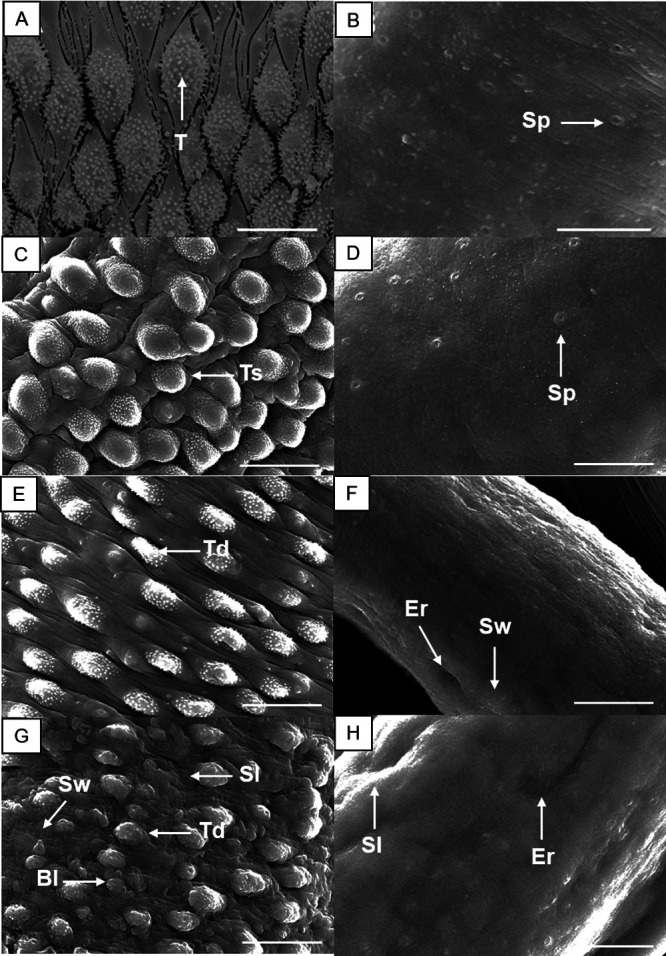
Scanning electron microscopy of adult S. mansoni following incubation with nifuroxazide (NFZ). Control male parasite showing intact tubercles (T) and spines on the surface (A), and control female worm (B) showing the sensory papillae (Sp). Schistosomes were exposed to NFZ at 12.5 μM (C and D), 25 μM (E and F), and 50 μM (G and H). Male (A, C, E, and G) and female (B, D, F, and H) schistosomes. The dorsal tegumental surface shows tubercle shortening (Ts), swelling (Sw), erosion (Er), sloughing (Sl), blisters (Bl), and tubercle disintegration (Td). Images were captured using a JEOL SM 6460LV scanning electron microscope after 72 h of incubation. Scale bars: 10 μm.

Scanning electron microscopy examination revealed a normal tegument topography of control group schistosomes, with the typical preservation of tubercles and spines (Fig. 2A and B). In contrast, NFZ at lethal concentrations (50 and 12.5 μM) caused morphological alterations in the tegument of adult schistosomes, which were more pronounced in male (Fig. 2C, E, and G) than in female helminths (Fig. 2D, F, and H). Male parasites exhibited substantial tegumental damage throughout the whole body, with the tubercles and spicules losing their natural shape.

### NFZ did not exhibit cytotoxicity on Vero cells.

A cytotoxicity assay was performed to evaluate the 50% cytotoxic concentration (CC_50_) and the selectivity index (SI; ratio between the CC_50_ values for the cells and the EC_50_ values for schistosomes). As shown in Table 1, NFZ did not exhibit toxicity at the maximum concentration tested (200 μM), with an SI of >10.

### NFZ reduces worm burden and egg production *in vivo*.

Considering the *in vitro* results, we investigated the efficacy of NFZ and PZQ administered orally (400 mg/kg of body weight) to mice harboring either juvenile (prepatent infection) or adult (patent infection) S. mansoni. Results were compared with that of the control infected but untreated animal. Of note, all drugs were well tolerated, and all mice survived until the end of the experimental work.

**FIG 3:**
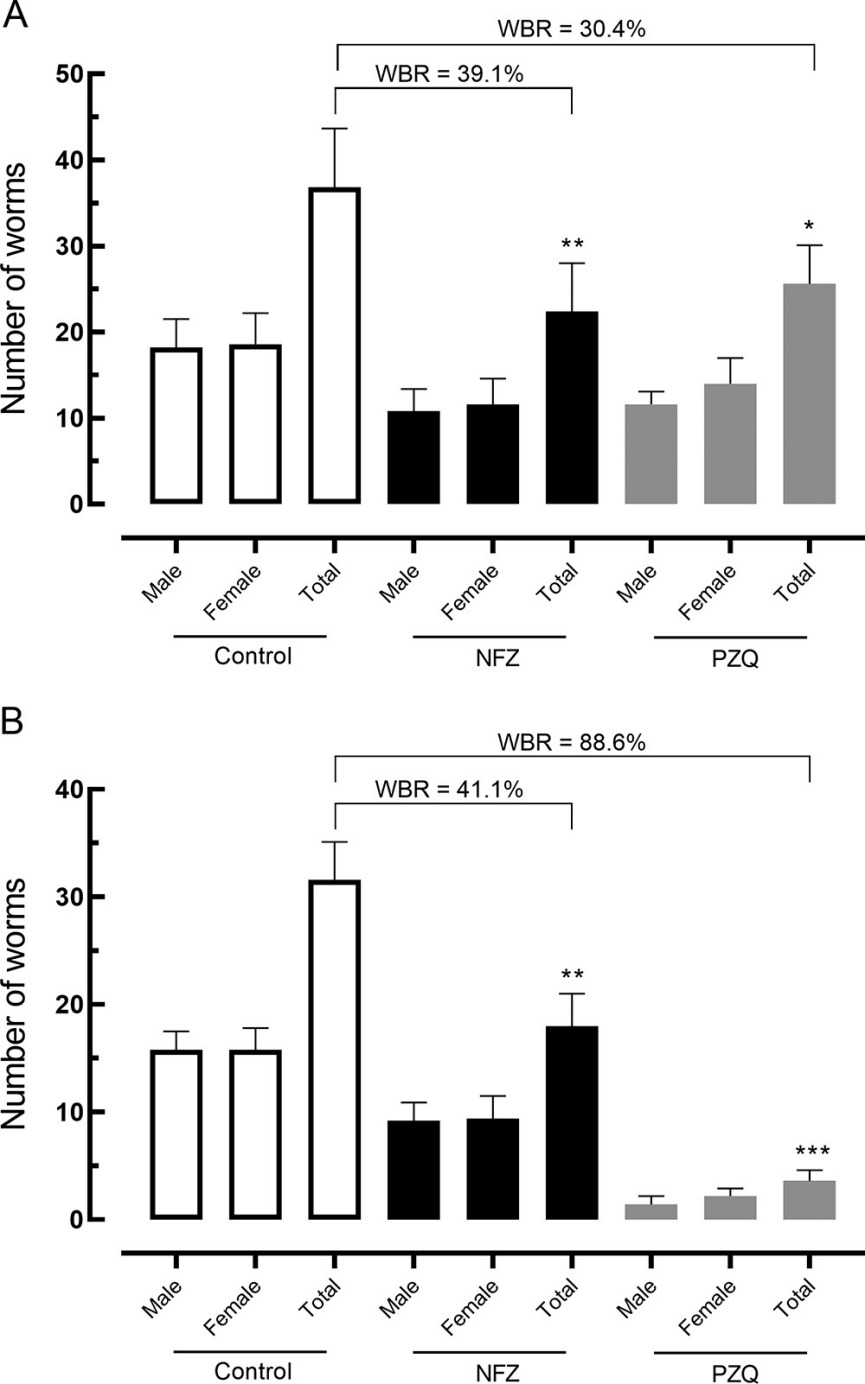
Effect of nifuroxazide (NFZ) and praziquantel (PZQ) on the parasite burden of mice with prepatent (A) and patent (B) Schistosoma mansoni infection. Vehicle (control), NFZ, and PZQ (400 mg/kg, single dose) were administered at 21 days (A) or 42 days (B) postinfection by oral gavage. On day 56 postinfection, all rodents were euthanized and parasite burdens were determined. Data are presented as the mean ± SD from five animals (*n *= 5 per group). *, *P < *0.05; **, *P < *0.01; ***, *P* < 0.001; *P* values were compared with infected untreated control. WBR, worm burden reduction.

Total worm burden reductions and male and female burden reductions following a single oral dose of NFZ and PZQ are presented in Fig. 3. NFZ significantly reduced worm burden (~40%; *P < *0.01) in both prepatent and patent S. mansoni infections compared with control S. mansoni-infected mice (Fig. 3A and B). Administration of PZQ led to a low, but significant, reduction in the number of parasites in animals with prepatent infection (30.4%; *P < *0.05) (Fig. 3A). In contrast, a high total worm burden reduction of 88.6% (*P < *0.001) was observed when the mice were treated with PZQ (Fig. 3B).

**FIG 4:**
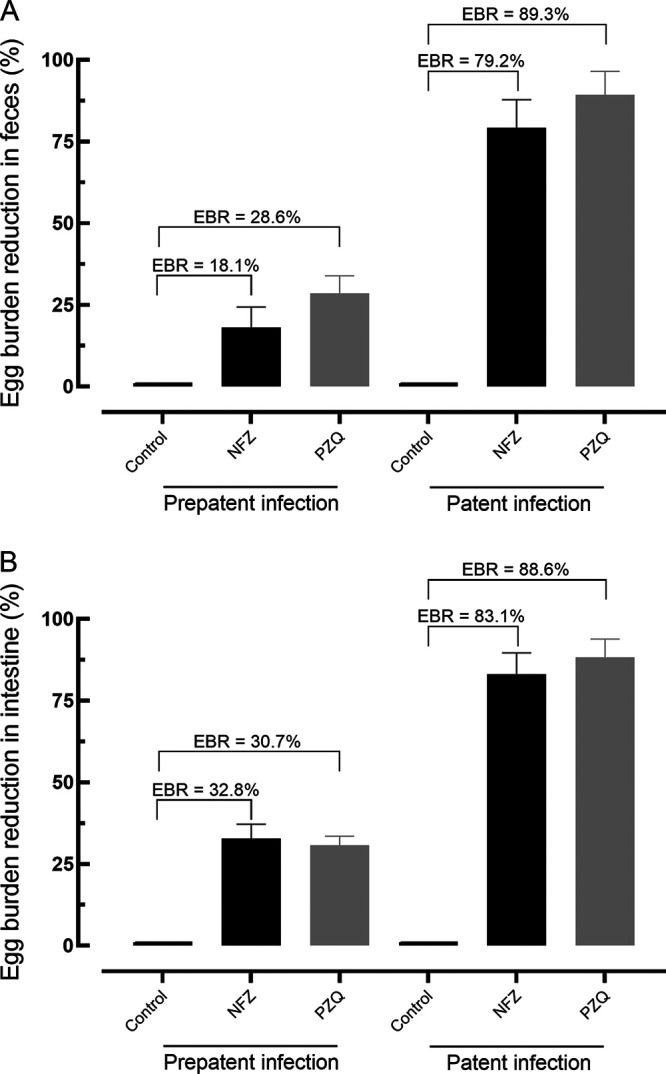
Effect of nifuroxazide (NFZ) and praziquantel (PZQ) on the egg burden in feces (A) and intestine (B) of mice with prepatent and patent Schistosoma mansoni infection. Vehicle (control), NFZ, and PZQ (400 mg/kg, single dose) were administered at 21 days (prepatent infection) or 42 days (patent) postinfection by oral gavage. On day 56 postinfection, all rodents were euthanized, and egg burden was determined by Kato Katz technique (egg in the feces) and oogram examination (immature eggs in the intestine). Data are presented as the mean ± SD from five animals (*n *= 5 per group). EBR, egg burden reduction.

The effect of NFZ on egg production was evaluated using the Kato-Katz method (eggs in the feces) and the oogram technique (eggs in the intestine). Results are summarized in Fig. 4.

For fecal examination, the oral dose of NFZ led to a high reduction in the number of eggs (~80%; *P < *0.001) in the patent infection, whereas NFZ did not cause a significant reduction in the fecal egg burden of animals with prepatent infection. For comparison, PZQ achieved a fecal egg reduction of 89.3% and 28.6% in animals with patent and prepatent S. mansoni infection, respectively (Fig. 4A).

Regarding the egg burden in the intestine, a single dose of NFZ was able to reduce the number of immature eggs by 32.8% and 83.1% in mice with prepatent and patent infection, respectively. For comparison, PZQ resulted in a reduction of the egg burden by 30.7% and 88.6% during the prepatent and patent periods, respectively (Fig. 4B).

### Target fishing studies have identified protein targets for NFZ.

Reverse docking studies were also performed to propose potential macromolecular targets for the action of NFZ. Its potential to produce reactive oxygen species employing nitroreductase catalysis is well known, but it is also known that more specific mechanisms of action, involving proteins as targets, can also take place (22, 23). To study potential receptors for NFZ, its structure was optimized, and its potential energy was calculated together with ChelpG charges by the HF/6-31G* computational method and subjected initially to pharmacophore screening using PharmMapper (Table 2).

**TABLE 2:** List of protein targets predicted for nifuroxazide using the reverse pharmacophore approach with PharmMapper and similarity by Basic Local Alignment Search Tool

PharmMapper results					BLAST results				
Target		No. of features	Fit score	Z score	Corresponding target in S. mansoni	Max score^*a*^	Query cover (%)^*b*^	E value^*c*^	Identity (%)^*d*^
PMID	Name								
2VTI	Cell division protein kinase 2	7	4.674	5.40965	Serine/threonine kinase	374	98	4e-128	60.54
1U4O	l-lactate dehydrogenase	5	3.897	4.31237	Putative l-lactate dehydrogenase	132	84	8e-35	28.25
1WBS	Mitogen-activated protein kinase 14	7	3.969	3.79749	Serine/threonine kinase	305	95	1e-100	45.43
1J4H	Peptidyl-prolyl *cis*-*trans* isomerase FKBP1A	9	4.173	3.50129	Putative immunophillin FK506 binding protein FKBP12	152	99	3e-48	66.98
1G45	Carbonic anhydrase 2	5	3.72	3.4792	Putative carbonic anhydrase II	196	98	3e-61	40.23
1CXV	Collagenase 3	7	3.906	3.05545	Matrix metallopeptidase-7 (M10 family)	132	95	2e-36	45.78

a Max score, the highest alignment score calculated from the sum of the rewards for matched nucleotides or amino acids and penalties for mismatches and gaps.

b Query cover, the percentage of the query sequence (our specimen) that overlaps the reference sequence.

c E value, the number of expected hits of similar quality (score) that could be found just by chance.

d Identity, the number of matching bases over the number of alignment columns (64).

Unfortunately, *Schistosoma* molecular targets are uncommon among the well-described targets or pharmacophores screened by the PharmMapper database. For this reason, none of the retrieved targets corresponded directly to any Schistosomatidae protein but rather corresponded to humans and other more complex organisms. A search for local similarity between protein sequences was then performed to find homologous proteins among the known proteome of *Schistosoma* species, employing the basic local alignment search tool (BLAST). Table 2 lists the targets for which any proteome correspondence was found. Among protein targets predicted, serine/threonine kinases (STK) appeared twice among the results, when presenting the highest values of max scores, indicating that they were the best alignments obtained. Moreover, expected values (E values) were also the lowest among the results, which translates to their lower probability of arbitrary alignments. Matrix metallopeptidase-7 from S. mansoni and three putative targets (l-lactate dehydrogenase [LDH], immunophilin FK506 binding protein FKBP12, and carbonic anhydrase II) were also predicted to interact with NFZ.

## DISCUSSION

The increasing recognition of the burden imposed by helminthiasis, associated with the limited therapeutic arsenal, has led to initiatives and strategies to research and develop new drugs for the treatment of schistosomiasis (9, 10). One of these strategies is drug repurposing, which considers low-risk compounds with potentially reduced costs and shorter time for development (24). NFZ, a well-known and often used antidiarrheal medicine, has been explored for its antiparasitic capabilities against parasites, such as *Leishmania* and *Trypanosoma* species (20). To our knowledge, in this study, we demonstrate for the first time that the antibacterial drug NFZ exhibits anthelmintic properties against the flatworm S. mansoni *in vitro* and in an animal model of schistosomiasis, for both prepatent and patent schistosome infections.

*In vitro* experiments revealed concentration-dependent, detrimental effects of NFZ on S. mansoni worm pairs, with male parasites being more susceptible to NFZ than females. Although this difference in drug sensitivity concerning the sexes of schistosomes was unknown, several antischistosomal agents are known to act more on male than on female worms (25–28). In addition, it is known that PZQ is more effective in male than in female worms (29), corroborating the results obtained in this study. NFZ also had detrimental effects on egg production *in vitro*, which is significant because PZQ, when administered at sublethal concentrations, is considerably less effective at reducing the egg count.

The tegumental outer surface of S. mansoni is of crucial importance for parasite survival, thus being a prime target for antischistosomal drug discovery studies (14, 18). Indeed, several studies have shown morphological alterations in schistosomes tegument following exposure to antiparasitic compounds, including PZQ (e.g., reference 28). Phenotypic studies using scanning electron microscopy revealed that NFZ induced substantial tegumental alterations in schistosomes, supporting the hypothesis that the parasite surface is one of the main targets of antischistosomal agents. These results regarding morphological changes could be due to the high lipophilicity of NFZ; consequently, this drug can cross the tegument of schistosomes and reach its molecular target(s).

To obtain further evidence for the importance of NFZ as an antischistosomal compound, NFZ was evaluated in both early and patent S. mansoni infection models in mice using a single oral dose of 400 mg/kg, following the protocol recommended for experimental schistosomiasis in a murine model (19). The treatment with NFZ revealed significant worm burden reductions in rodents with either prepatent or patent infection. It should be noted that PZQ treatment exerts varied cure rates (70 to 90%) (30), but it is concerning that some infections in humans and various other species of animals appear to be refractory to treatment (31, 32). Importantly, it is known that PZQ has low efficacy against juvenile parasites (prepatent infection) (33). Comparatively, oral treatment with NFZ is more effective in early infection than PZQ. These data suggested the advantage of using NFZ instead of PZQ in immature S. mansoni stages.

The efficacy of NFZ therapy in S. mansoni egg production was also evaluated. Interestingly, similar to PZQ, an oral dose of NFZ led to a high reduction in the number of eggs in patent infection (~80%). Since NFZ did not cause a high reduction in the number of worms in patent S. mansoni infections, these results show that the drug interferes directly with egg production, similar to what was observed in our *in vitro* experiments. Consistent with the results obtained, a significant egg burden reduction, even when worm reduction was low, has been reported with other antischistosomal compounds (34). Collectively, considering that egg production is important for both disease transmission and pathogenesis (1), the negative impact on parasite egg laying is of interest when seeking drugs with anthelmintic properties (9, 11).

The maximum plasma concentration (*C*_max_) for NFZ is achieved typically within 2 to 3 h after oral administration. The specific *C*_max_ can vary depending on factors such as the formulation, dose, and individual patient characteristics, but information regarding the *C*_max_ of NFZ is scarce. In mice treated intraperitoneally with NFZ at a dose of 15 mg/kg three times per week for 2 weeks, the mean levels of NFZ in the plasma corresponded to 8.5 μM (35), a concentration close to the EC_50_ described in this study. In rats, about 17% of orally administered NFZ reaches systemic circulation (36). The effectiveness of oral NFZ in S. mansoni-infected animals supports this concept, suggesting that NFZ may be effective in a clinically relevant dose range. In fact, in clinical trials using adult patients, NFZ has been used at a dose of 800 mg per day (37, 38). However, further studies are necessary to evaluate the pharmacokinetic properties and antischistosomal role of orally administered NFZ.

Literature on the mechanism of action of NFZ in bacteria is limited. NFZ exerts bactericidal or bacteriostatic effects, depending on concentration (39). The drug disrupts pathogenic metabolism by interfering with the nucleic acids of pathogens (21). In cancer cells, NFZ has been reported as a potent inhibitor of the signal transducer and activator of transcription 3 (STAT3) (40). To analyze the mechanism of action of NFZ on schistosomes, we performed target fishing studies. STK was one of the main targets of the NFZ, and its activity has been associated with the surface molecules of schistosomes (41). It is known that STKs are enzymes widely found in Apicomplexa (42) but are still under study in helminths (43). They seem to be related to sexual development and maturation and their modulation could affect oogenesis, spermatogenesis, and oviposition which, in the case of S. mansoni, can halt the pathological progress.

An interesting target that has been pointed out is the already characterized matrix metallopeptidase-7 from S. mansoni. This protein is from the M10 family and, as such, is probably involved in the degradation of the extracellular matrix when it is required, such as in tissue remodeling and embryonic development. It can break down proteoglycans, fibronectin, elastin, and casein to achieve this matrix decomposition. In S. mansoni, this enzyme is involved in collagen degradation, which allows the eggs to circulate within the host organism. McCrudden and Iredale (44) showed that an imbalance between the action of degradative metalloproteinases and tissue inhibitors of metalloproteinases leads to liver fibrosis. Moreover, Singh and coworkers (45) proposed that the same imbalance could take place in S. mansoni egg-induced fibrosis, which suggests that the worm metalloproteinase develops a role in egg delivery throughout the host body. Modulation of this enzyme could thus lead to an interesting way of helping the resorption of the eggs by the host tissues.

Three of the correspondents found (LDH, immunophillin FK506 binding protein FKBP12, and carbonic anhydrase II) are putative proteins. In other words, these proteins are probable proteins still to be isolated and characterized within the worm proteome, and their existence is attested only by similarity to genome sequences. NFZ acts by interfering with the activity of enzymes, such as NADH dehydrogenase and certain enzymes involved in electron transport chains within bacterial and protozoal cells (21, 46). By disrupting these key metabolic processes, NFZ disrupts the energy production and survival of the microorganisms (20). Since the parasitic stages of *Schistosoma* depend on anaerobic energy metabolism, LDH appears to be an interesting target for the development of novel anthelmintic agents. Recent studies have acknowledged the potential of LDH as a target for antiparasitic drug development (47). Carbonic anhydrase II is another putative enzyme that our group has already identified as a potential target for the *N*-acylhydrazone scaffold (43). This enzyme is supposed to be involved in the osmotic balance and acid-base homeostasis of the worms, and it is located mainly on the tegument surface.

It is important to note that the precise mode of action of NFZ may vary depending on the specific microorganism being targeted (48). Furthermore, research on the mechanism of action of NFZ is ongoing, and further studies may provide additional insights into its exact molecular interactions and pathways (49). All the targets discussed here must be validated experimentally, but the target fishing studies can, at least, point out some directions toward understanding the mechanism of action.

Many studies have revealed that NFZ is well tolerated and safe (38, 50). Advantageously, even at high dosages, NFZ does not affect the integrity of intestinal microbiota (50). In this study, we provide important information regarding the antiparasitic activities of NFZ against S. mansoni. We demonstrated that NFZ affected parasite viability and egg production, and it induced severe tegumental damage in schistosomes. In an animal model of schistosomiasis, we further demonstrate that NFZ is orally effective in both prepatent and patent infections. Moreover, target fishing investigations have identified molecular targets for NFZ, a finding that needs to be validated in experimental target deconvolution studies. Therefore, these results implied that NFZ might be a potential therapeutic candidate for the treatment of schistosomiasis.

## MATERIALS AND METHODS

### Drugs and reagents.

RPMI 1640 medium, Dulbecco’s modified Eagle medium (DMEM), heat-inactivated fetal calf serum, and penicillin G-streptomycin solutions (10,000 U/mL penicillin G sodium salt and 10 mg/mL streptomycin sulfate) were obtained from Vitrocell (Campinas, SP, Brazil). HEPES buffer, dimethyl sulfoxide (DMSO), and thiazolyl blue tetrazolium bromide (MTT) were purchased from Sigma (St. Louis, MO). Praziquantel was kindly provided by Ecovet Indústria Veterinária Ltda (São Paulo, SP, Brazil). NFZ was synthesized as describe in the published protocols (22, 51). In all *in vitro* experiments, compounds were solubilized in DMSO.

### Animals, parasites, and cells.

The life cycle of S. mansoni (BH strain) is maintained by routine passage through Biomphalaria glabrata snails and Swiss mice at Guarulhos University (UNG, Guarulhos, SP, Brazil). Both rodents and snails were kept at 25°C and 50% humidity with an artificial 12-h/12-h day/night cycle and provided with water and food *ad libitum*. The 4-week-old mice were infected subcutaneously with S. mansoni cercariae, which were collected from S. mansoni-infected snails (39, 52).

Vero cells (monkey kidney epithelial cells) were obtained from the American Type Culture Collection (ATCC CCL-81; Manassas, VA). Cells were cultured in DMEM containing 2 mM l-glutamine, antibiotics (100 U/mL penicillin and 100 μg/mL streptomycin), and 10% heat-inactivated fetal bovine serum and were kept at 37°C in a humidified atmosphere containing 5% CO_2_. Cells were maintained in 25-cm^2^ culture flasks (Corning, Tewksbury, MA) and harvested using 0.25% trypsin in 0.2 g/L EDTA solution (28).

### *In vitro* antiparasitic assay.

Adult S. mansoni were collected from mice by dissection at 42 days postinfection and were maintained in RPMI 1640 culture medium supplemented with 5% fetal calf serum, 100 U/mL penicillin, and 100 μg/mL streptomycin at 37°C and 5% CO_2_. Compounds were diluted to 50 μM in supplemented RPMI medium in 24-well plates (Corning, New York, NY), to which one worm of each sex was added per well (53, 54). Each concentration was tested at least in triplicate, and the experiments were repeated three times. Parasites incubated with drug-free DMSO (0.5%) served as a control. The egg output and viability of adult schistosomes were assessed via microscopic readout at 1, 24, 48, and 72 h (33) using a Motic AE2000 inverted microscope (Vancouver, Canada) equipped with a Motic ultrahigh definition (UHD) camera and with a 48-inch 4K-UHD monitor system (LG Electronics, Taubaté, SP, Brazil) (55). The death of adult schistosomes was defined as no movement observed for at least 1 to 2 min of examination, whereas parasites with any body movement were considered viable (28). The percentage of viable parasites was calculated considering schistosomes exposed to compounds versus control worms.

### Scanning electron microscopy investigation.

Scanning electron microscopy studies were performed as described previously (26, 56). Briefly, schistosomes (treated and control groups) were fixed in 2.5% glutaraldehyde, and mounted specimens were coated with gold sputter (Denton Vacuum LLC, Moorestown, NJ) and photographed using a JEOL JSM-6460LV scanning electron microscope (Tokyo, Japan).

### *In vitro* cytotoxicity assay.

The MTT assay was used to evaluate the cytotoxic activity as described previously (57). Briefly, cells were plated in 96-well plates (TPP Techno Plastic Products AG, Trasadingen, Switzerland) at a density of 2 × 10^3^ cells/well in the presence of NFZ at different concentrations (starting at 200 μM and following a 3-fold dilution series) for 72 h at 37°C and 5% CO_2_. After the addition of MTT solution, the cells were maintained at 37°C for 4 h. Absorbance was measured at 595 nm using a spectrophotometer (Epoch, BioTek Instruments, Winooski, VT), and the percentage of viable cells was determined concerning the control wells. At least two independent experiments in triplicate were carried out for each test compound. The selectivity indices (SIs) were calculated by dividing the 50% cytotoxic concentration (CC_50_) obtained on cells with 50% effective concentration (EC_50_) values determined on schistosomes (58).

### *In vivo* studies in S. mansoni-infected mice.

*In vivo* studies were performed according to drug discovery programs for schistosomiasis (59). Animal studies are reported in compliance with the National Centre for the Replacement and Refinement & Reduction of Animals in Research (NC3Rs) Animal Research: Reporting of *In Vivo* Experiments (ARRIVE) guidelines. For experimental protocols, 30 Swiss mice, at 3 weeks old, were infected subcutaneously with 80 S. mansoni cercariae each. Animals were then divided randomly into six experimental groups (five mice per group), and drugs (NFZ and PZQ) or a vehicle (2% ethanol in water) was administered for 21 days (immature parasite, prepatent infection) or 42 days (adult parasite, patent infection) postinfection by oral gavage using a single oral dose of 400 mg/kg (28, 60). On day 56 postinfection, animals in all groups were euthanized using CO_2_; worms were picked, sexed, and counted; and the worm burden reduction was calculated (61). Therapeutic efficacy was also based on the technique of qualitative and quantitative oograms in the intestine, as well as the Kato-Katz method for quantitative fecal examination, as reported previously (62).

All parameter (worm counts, quantitative and qualitative oogram, and quantitative fecal examination) measurements were performed by different people (by at least two different investigators). To eliminate bias in interpretation, the manipulators of the experiments were not the same researchers as the data analysts (16).

### Target fishing studies.

The NFZ structure was constructed using GaussView 5.0 and optimized by employing the *ab initio* method HF/6-31G* with energy calculated considering ChelpG charges (Gaussian 09W). A mol2 file was constructed within an open babel converter, considering ChelpG as the atomic charges, and was input into the PharmMapper Web-based software (63) for pharmacophore screening. Simulation conditions were adjusted to “generate conformers,” employing the charges and initial conformation from the input file, screening the “All Targets” pharmacophore database, and “retrieving the best 300 results.” The top 25 results were analyzed by their Z score and FitScore values, and only those with the highest values (<2.5) were considered for the Basic Local Alignment Search Tool (BLAST) study (45). Each target was then aligned with known and putative proteins from the Schistosomatidae family database, through their FASTA files (protein primary sequences), employing the BLAST Web-based tool (64).

### Statistical analysis.

Statistical analyses were performed using Graph Pad Prism software 8.0 (San Diego, CA). EC_50_, EC_90_, and CC_50_ values were calculated using sigmoid dose-response curves (32). For the experimental analysis of animal studies, the nonparametric Kruskal Wallis test was applied to compare the control group with the treated group (34). The level of statistical significance was set to a *P* value of <0.05. The data and statistical analysis comply with the recommendations on experimental design and analysis in the pharmacology field (14).

### Ethical approval.

Animal studies are reported in compliance with the ARRIVE guidelines. The protocol for experimental design was reviewed and approved by the Committee for the Ethical Use of Animals in Experimentation of Guarulhos University (Guarulhos, SP, Brazil; protocol identifier [ID] 47/20) in conformity with the Brazilian law for Guidelines for Care and Use of Laboratory Animals.

### Data availability.

The raw data that support the findings of this study are available from the corresponding author upon reasonable request.
